# Bullöse Mycosis fungoides – ein klinischer Indikator für einen aggressiven Krankheitsverlauf

**DOI:** 10.1007/s00105-024-05399-4

**Published:** 2024-08-09

**Authors:** Ana-Lee Gerdes, Julia Hyun, Alexander Kreuter, Jörg Schaller, Uwe Hillen, Alena-Lioba Michalowitz, Valentina Müller

**Affiliations:** 1 Klinik für Dermatologie, Venerologie und Allergologie, Helios Klinikum St. Johannes Klinik Duisburg, Duisburg, Deutschland; 2 Klinik für Dermatologie, Venerologie und Allergologie, Helios Klinikum St. Elisabeth Klinik Oberhausen, Universität Witten/Herdecke, Josefstr. 3, 46045 Oberhausen, Deutschland; 3 Dermatopathologie Essen, Essen, Deutschland

**Keywords:** Kutanes T-Zell-Lymphom, Blasen, Transformation, Krankheitsprogression, Cutaneous T-cell lymphoma, Blisters, Transformation, Disease progression

## Abstract

Die Mycosis fungoides (MF) ist das häufigste kutane T‑Zell-Lymphom (CTCL). Die bullöse Form gilt als eine ihrer zahlreichen Varianten. In der Literatur sind nur wenige Fälle dieser seltenen Entität beschrieben. Wir berichten von einem männlichen Patienten mit fulminantem Verlauf einer bullösen MF, die aufgrund der infausten septischen Gesamtkonstellation innerhalb weniger Wochen zum Exitus letalis führte.

## Abstract (en)

Mycosis fungoides (MF) is defined as the most common cutaneous T‑cell lymphoma (CTCL). The bullous form is considered one of its numerous variants. Only a few cases of this rare entity have been described. We report the case of a man with an aggressive course of bullous MF, which led to lethal outcome within a few weeks due to a fulminant sepsis.

## Anamnese

Ein 92-jähriger, bis dato noch sehr rüstiger Patient, war aufgrund einer akuten Verschlechterung seines Allgemeinzustandes mit Gliederschmerzen, Schüttelfrost sowie febriler Körpertemperatur notfallmäßig in unserer dermatologischen Ambulanz vorstellig geworden. Eine aktive Infektion mit SARS-CoV‑2 konnte molekularbiologisch gesichert werden. Der Patient war mit einer erstmalig 2018 diagnostizierten Mycosis fungoides im Stadium IIB in domo bereits bekannt. Therapeutisch waren in der Vergangenheit mehrere Zyklen einer extrakorporalen Photopherese, Creme-PUVA (Psoralen und UVA) und diverse Systemtherapien (u. a. Roferon, Bexaroten und Neotigason) sowie eine Radiatio (Gesamtdosis 24 Gy) einer Tumorläsion am linken Oberschenkel erfolgt. Hierunter war jeweils eine passagere Befundstabilisierung zu erreichen.

In den letzten Wochen hatte der Patient jedoch rasch progrediente bullöse Plaques und Nodi am gesamten Integument entwickelt, die nachfolgend ulzerierten. Zeitgleich war ein stetiger Gewichtsverlust bei anamnestisch weiterhin vorhandenem Appetit aufgetreten. Akute kardiale, pulmonale oder gastrointestinale Beschwerden wurden verneint. Nebenbefundlich bestanden eine arterielle Hypertonie, absolute Arrhythmie bei Vorhofflimmern, chronisch obstruktive Lungenerkrankung (COPD), chronische Niereninsuffizienz bei Zustand nach Nephrektomie aufgrund eines Nierenzellkarzinoms rechts, periphere arterielle Verschlusskrankheit (pAVK) sowie ein Diabetes mellitus Typ 2 mit diabetischer Polyneuropathie. Die stationäre Aufnahme erfolgte bei SARS-CoV-2-Infektion und akuter Verschlechterung der Mycosis fungoides mit nun neu aufgetretener Eruption bullöser Läsionen.

## Hautbefund

**Abb. 1:**
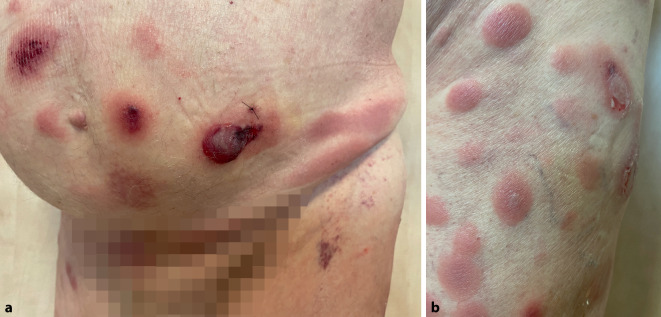
Klinischer Befund bei Erstvorstellung. Abdominell, am Mons pubis (**a**) sowie am rechten Oberschenkel ventrolateral (**b**) imponieren initial disseminierte, indurierte, erythematöse Plaques und Nodi mit teils zentraler, schlaffer Blasenbildung

**Abb. 2:**
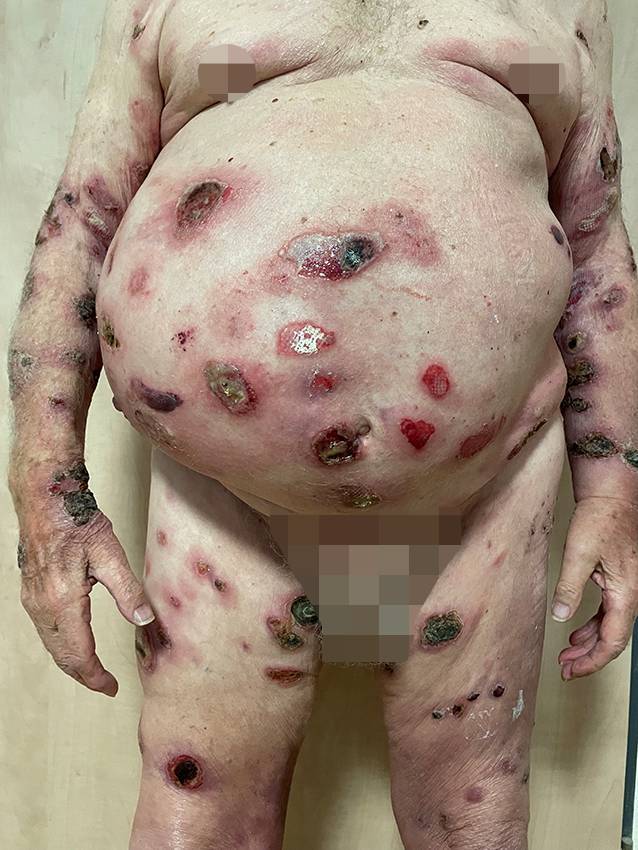
Fulminanter Verlauf ca. 4 Wochen nach Erstvorstellung mit einem Mischbild aus rundlich-ovalen, teils fibrinösen, teils konfluierenden Ulzerationen mit teils zentraler Nekrosebildung, schlaffen Blasen sowie erythematösen Plaques

Initial imponierte stammbetont sowie auch an beiden streck- und beugeseitigen, oberen und unteren Extremitäten ein Mischbild aus ca. 3 cm x 2 cm durchmessenden, teils flächig konfluierenden, infiltrierten Nodi und Plaques mit teils zentraler, schlaffer Blasenbildung mit gelblich-trübem Inhalt sowie unscharf begrenzten, anulär anmutenden Erythemen und Plaques (Abb. 1). Im weiteren Verlauf, nach ca. 4 Wochen, konfluierten die Effloreszenzen zu ca. 10 cm durchmessenden, nässenden, teils fibrinbelegten Ulzerationen, eitrig sezernierenden Plaques sowie Nekrosen (Abb. 2). Die Schleimhäute zeigten sich blande. Der Lymphknotenstatus war unauffällig.

## Diagnostik

**Abb. 3:**
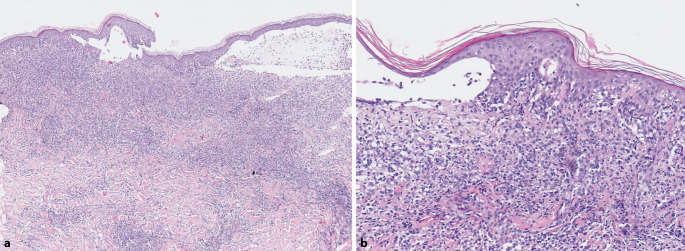
Histopathologisch zeigt sich ein für die Mycosis fungoides typischer Epidermotropismus (**a**). Pautrier-Mikroabszess mit subepidermaler Blasenbildung sowie Spongiose (**b**). Hämatoxylin-Eosin-Färbung, Originalvergrößerung 100:1

**Abb. 4:**
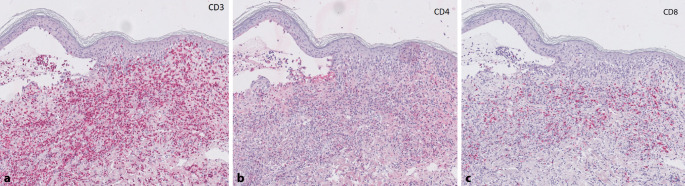
Immunhistochemische Untersuchung mit CD3 (**a**). Schwach positive Färbung mit CD4 (**b**). Deutlich geringere Positivität des Infiltrates auf CD8 (**c**), Originalvergrößerung 100:1

Histopathologisch fand sich in der oberen und mittleren Dermis ein dichtes Infiltrat mittelgroßer, teils atypischer Lymphozyten mit deutlichem Epidermotropismus (Abb. 3). Des Weiteren zeigte sich im Bioptat eine subepidermale Blasenbildung. Spezifische Immunfluoreszenzphänomene waren in der direkten Immunfluoreszenz nicht nachweisbar. Auch serologisch bestand bei negativem Nikolski-Phänomen kein Anhalt für eine blasenbildende Autoimmundermatose. In den vorangegangenen immunhistochemischen Befunden zeigte sich das Infiltrat deutlich positiv für CD3 und CD4, hingegen deutlich weniger positiv für CD8 (Abb. 4). Immunhistochemisch dominierten in der aktuellen Verlaufskontrolle bei immunphänotypisch verminderter CD4/CD8-Ratio kleine CD3-positive, atypische T‑Lymphozyten bei nahezu vollständigem Verlust von CD4, CD8 und CD30 sowie fehlender Expression von CD2 und CD56. Partiell wurden CD45R0 und CD45RA exprimiert. Beta F1 als Hinweis für die Expression der T‑Zell-Rezeptor-alpha-beta-Kette war nachweisbar. In der Ki67-Färbung zeigte sich bei 60 % der Lymphozyten eine signifikant hohe proliferative Aktivität. In der PGM1-Färbung waren verstreut Histiozyten zu sehen. Die atypischen Lymphozyten zeigten eine Expression von TIA‑1 sowie partielle Expression von Granzyme B als Ausdruck eines zytotoxischen Phänotyps. Molekulargenetisch ergab sich keine klonale Genumlagerung des T‑Zell-Rezeptors-gamma-Gen-Locus. In der T‑Zell-Rezeptors-beta-Gen-Analyse fanden sich vereinzelt reproduzierbare Peaks.

In Gesamtschau der Befunde konnten wir eine Transformation der vorbekannten Mycosis fungoides in eine bullöse Variante diagnostizieren.

Im Blasenabstrich ließen sich *Staphylococcus aureus* sowie *Klebsiella pneumoniae* nachweisen. In den Blutkulturen konnte *Staphylococcus aureus* angezüchtet werden. Hinsichtlich der Methicillin-resistenten *Staphylococcus-aureus*-Bakteriämie ließ sich sowohl echokardiographisch eine Endokarditis als auch magnetresonanztomographisch eine Spondylodiszitis ausschließen. Zudem zeigte sich ein akutes bzw. im weiteren Verlauf terminales Nierenversagen unter abdomensonographischem Ausschluss einer postrenalen Genese. Im Verlauf entwickelte der Patient eine Sepsis mit zusätzlichen Pleuraergüssen beidseits und einem neu aufgetretenen, herdförmigen Infiltrat im rechten Oberlappen, a. e. im Sinne eines septischen Streuherdes. Lymphomsuspekte Läsionen konnten bei unauffälliger sonographischer Darstellung der zervikalen, axillären sowie inguinalen Lymphknoten und computertomographisch sowohl thorakal, abdominell als auch im kleinen Becken ausgeschlossen werden.

## Therapie und Verlauf

Aufgrund der akuten Allgemeinzustandsverschlechterung leiteten wir bei erhöhten Infektparametern und anamnestisch angegebener Penicillinallergie zunächst eine kalkulierte, intravenöse Antibiose mittels Clindamycin 600 mg 3‑mal täglich ein, die wir bei Nachweis von *Staphylococcus aureus* in den Blutkulturen auf Cefazolin 2 g 3‑mal täglich eskalierten. Supportiv erhielt der Patient unter Berücksichtigung der SARS-CoV-2-Infektion Paracetamol 1 g i.v. bis zu 3‑mal täglich. Die Wundversorgung erfolgte täglich mittels Polyhexanid-basiertem Hydrogel, Silikon-beschichteten Wunddistanzgittern auf den Ulzerationen und Dialkylcarbamoylchlorid-beschichteten Wundauflagen im Bereich der Nekrosen. Eine Nekrosektomie war unter Berücksichtigung der großflächigen nekrotischen Ausdehnung aufgrund des Risikos eines hierdurch getriggerten, fulminanten Verlaufes der septischen Situation vorerst nicht möglich. Bei Nachweis der Transformation der Mycosis fungoides in eine bullöse, hochakute Form planten wir nach Vorstellung des Falls in unserem interdisziplinären Tumorboard eine Low-dose-Systemtherapie mit Gemcitabin 1000 mg/m^2^ Körperoberfläche intravenös, konnten diese aufgrund der septischen Gesamtkonstellation jedoch nicht initiieren [2]. Trotz Flüssigkeitssubstitution und Pausierung nephrotoxischer Medikamente verblieb der Patient im akuten Nierenversagen. Bei unzureichendem klinischem und laborchemischem Ansprechen ergänzten wir die Antibiose intravenös um Meropenem 1 g 2‑mal täglich. Hinsichtlich der progredienten, peripheren Ödeme und beidseitigen Pleuraergüsse erfolgte kardioprotektiv eine Negativbilanzierung unter Pausierung der Flüssigkeitssubstitution sowie intravenöser Gabe von Furosemid. Im weiteren Verlauf wurde seitens des Patienten jegliche weitere diagnostische, therapeutische sowie lebenserhaltende Maßnahme, wissentlich der Konsequenzen, abgelehnt. Infolgedessen trat der Exitus letalis bei fulminanter Sepsis ein.

## Diskussion

Die Mycosis fungoides (MF) gilt als häufigstes, primär kutanes T‑Zell-Lymphom mit indolentem Verlauf [1]. Üblicherweise verläuft die Erkrankung in 3 Stadien, dem Patch‑, Plaque- und Tumorstadium, die zeitlich nacheinander oder auch parallel auftreten können. Neben der klassischen Form wird in der Literatur eine Vielzahl an Subtypen beschrieben, die sich in ihren klinischen, histopathologischen und immunhistochemischen Eigenschaften vom klassischen Typ unterscheiden [3].

Eine absolute Rarität stellt die bullöse Mycosis fungoides dar [4]. Erstmals 1887 von Kaposi als „pemphigusähnliche MF“ beschrieben, zeigen sich klinisch typischerweise schlaffe oder pralle, vesikulobullöse Läsionen, die meist simultan mit Patches, Plaques und Tumoren oder selten de novo als Erstmanifestation auftreten. Die Effloreszenzen finden sich lokalisiert oder disseminiert stammbetont sowie an den oberen und unteren Extremitäten auf gesunder Haut oder entwickeln sich aus bereits vorhandenen MF-Läsionen [5, 6]. Der zugrunde liegende Pathomechanismus ist bislang ungeklärt. Beschrieben worden ist u. a. eine intraepidermale Blasenbildung durch das Zusammentreffen von Pautrier-Mikroabszessen, eine epidermale Degeneration aufgrund eines Epidermotropismus, eine ausgedehnte Spongiosis, ein Kohärenzverlust zwischen Basallamina und basalen Keratinozyten durch neoplastische Lymphozyten sowie der Einfluss von aus atypischen Lymphozyten freigesetzten Lymphokinen [4, 7]. Diskutiert wurde ebenso eine vermehrte Kolonisierung der Haut mit *Staphylococcus aureus* als Stimulanz für die Bildung bzw. klinische Verschlechterung eines CTCL [11]. Differenzialdiagnostisch abzugrenzen sind v. a. blasenbildende Autoimmundermatosen, aber auch die akute Kontaktdermatitis, Arthropodenreaktionen, Infektionen mit Staphylokokken oder Herpesviren und ein bullöses Arzneimittelexanthem [7, 8]. Zu den von Bowman et al. zur diagnostischen Abgrenzung entwickelten Kriterien zählen klinisch vesikulobullöse Läsionen, histologische Merkmale wie atypische lymphatische Zellen, Epidermotropismus und Pautrier-Mikroabszesse mit intraepidermalen oder subepidermalen Blasen, eine negative Immunfluoreszenz sowie Ausschluss einer anderen infektiologisch bedingten, medikamentös oder therapeutisch induzierten Genese [5]. In der Literatur existieren ca. 35 beschriebene Fälle dieser seltenen Entität. Die Diagnose der bullösen MF geht meist mit einem fulminanten Verlauf sowie einer schlechten Prognose einher [8]. Als ungünstiges prognostisches Zeichen ist das Auftreten der Blasen selbst zu betrachten, welches auch mit dem raschen Fortschreiten der Erkrankung korreliert [7]. Radiatio und Zytostatika haben sich gegenüber Lokaltherapien und sonstigen Systemtherapien als wirksame Therapieansätze erwiesen [4, 7]. Trotz der hierunter erzielten Teil- bzw. Vollremission ist eine hohe Rezidivrate beschrieben [9, 10]. Die Mortalitätsrate beträgt 50 % innerhalb von 12 Monaten nach Auftreten der Bullae [6, 8]. Als prognostisch ungünstiger und optionaler Triggerfaktor wird ebenso das Vorhandensein von Ulzerationen mit erhöhtem Sepsisrisiko betrachtet [4]. In diesem Sinne scheint auch die kutane Besiedlung oder Infektion mit *Staphylococcus aureus* ein möglicher Triggerfaktor für einen erschwerten Verlauf zu sein, wobei zugrunde liegende Mechanismen noch unbekannt sind [11]. Die genannten Aspekte zusammengenommen, führten letztlich auch bei unserem Patienten zu einem hochakuten Verlauf mit letalem Ausgang.

## Fazit für die Praxis

- Die bullöse MF ist eine seltene Variante, die durch eine schlechte Prognose gekennzeichnet ist.
- Eine häufige, oftmals letale Komplikation der bullösen MF ist die Sepsis.
- Die Therapie sollte rasch eingeleitet werden; Zytostatika sind aufgrund des raschen Therapieansprechens in Erwägung zu ziehen.

## References

[1] Willemze R, Cerroni L, Kempf W et al (2019) The 2018 update of the WHO-EORTC classification for primary cutaneous lymphomas. Blood 133(16):1703–1714 30635287 10.1182/blood-2018-11-881268 PMC6473500

[2] Blazejak C, Stranzenbach R, Gosman J et al (2022) Clinical Outcomes of Advanced-Stage Cutaneous Lymphoma under Low-Dose Gemcitabine Treatment: Real-Life Data from the German Cutaneous Lymphoma Network. Dermatology 238(3):498–506 34474414 10.1159/000517830

[3] Willemze R (2018) Mycosis fungoides variants-clinicopathologic features, differential diagnosis, and treatment. Semin Cutan Med Surg 37(1):11–17 29719015 10.12788/j.sder.2018.004

[4] Sato S, Okamoto O, Kawamoto M et al (2011) Bullous mycosis fungoides associated with an extensive ulcer and a severe leukemoid reaction. Dermatol Reports 3(3):e54 25386305 10.4081/dr.2011.e54 PMC4211505

[5] Bowman PH, Hogan DJ, Sanusi ID (2001) Mycosis fungoides bullosa: report of a case and review of the literature. J Am Acad Dermatol 45(6):934–939 11712043 10.1067/mjd.2001.117521

[6] Kamran B, Fatemeh M, Ahmadreza R et al (2008) Bullous mycosis fungoides: a case report. Dermatol Online J 14(2):11 18700114

[7] Kneitz H, Bröcker EB, Becker JC (2010) Mycosis fungoides bullosa: a case report and review of the literature. J Med Case Rep 4:78 20196875 10.1186/1752-1947-4-78 PMC2838916

[8] Nofal A, Alakad R, Ehab R et al (2021) Mycosis fungoides bullosa: An unusual presentation of a rare entity. JAAD Case Rep 18:82–88 34869813 10.1016/j.jdcr.2021.10.019 PMC8626627

[9] Porntharukcharoen S, Rutnin S, Rajatanavin N (2017) Large-Cell Transformed Mycosis Fungoides Coexisting with Mycosis Fungoides Bullosa: A Case Report and Review of the Literature. Case Rep Dermatol 9(3):243–248 29515392 10.1159/000484472 PMC5836154

[10] Xu S, Weiss E, Singh K et al (2023) A case of bullous lesions in cutaneous T‑cell lymphoma. JAAD Case Rep 35:90–93 37223111 10.1016/j.jdcr.2023.02.026 PMC10201206

[11] Chi MH, Kuo TT, Lu PH et al (2013) Coexistent bullous and pustular mycosis fungoides in a patient with staphylococcal sepsis. Int J Dermatol 52(1):79–83 22998643 10.1111/j.1365-4632.2011.05208.x

